# Association Between Rental Assistance Programs and Hemoglobin A_1c_ Levels Among US Adults

**DOI:** 10.1001/jamanetworkopen.2022.22385

**Published:** 2022-07-20

**Authors:** Andrew Fenelon, Kasia J. Lipska, Whitney Denary, Kim M. Blankenship, Penelope Schlesinger, Denise Esserman, Danya E. Keene

**Affiliations:** 1 School of Public Policy and Department of Sociology and Criminology, Penn State University, University Park, Pennsylvania; 2 Department of Internal Medicine, Yale School of Medicine, New Haven, Connecticut; 3 Department of Social and Behavioral Sciences, Yale School of Public Health, New Haven, Connecticut; 4 Department of Sociology, American University, Washington, DC; 5 Department of Biostatistics, Yale School of Public Health, New Haven, Connecticut

## Abstract

**Importance:**

Programs that provide affordable and stable housing, such as federal rental assistance, may be associated with improved mean blood glucose levels and related diabetes outcomes.

**Objective:**

To assess whether 2 different types of federal rental assistance programs are associated with glycated hemoglobin A_1c_ (HbA_1c_) levels among middle-aged and older US adults.

**Design, Setting, and Participants:**

This cohort study used data from the National Health and Nutrition Examination Survey (NHANES) linked with US Department of Housing and Urban Development records of rental assistance participation. Adults aged 45 years or older who were receiving 2 types of rental assistance (project-based housing or housing vouchers) at the time of the NHANES interview and those who would receive rental assistance within the subsequent 2 years (waitlist group) were included. Data were collected from January 1999 to December 2016 and analyzed in October 2021.

**Exposures:**

Rental assistance participation, including project-based housing (subsidized housing developments including public housing) and housing vouchers (tenant-based subsidies for private market housing).

**Main Outcomes and Measures:**

The primary outcome was continuous HbA_1c_ level, a common measure of blood glucose reflecting diabetes control. Linear regression was used to estimate the association between the 2 rental assistance programs and HbA_1c_ level. Logistic regression was used to assess the association between rental assistance programs and HbA_1c_ cut points (prediabetes: 5.7% to ≤6.5%; diabetes: >6.5%; uncontrolled diabetes: ≥9% [to convert to proportion of total Hb, multiply by 0.01]). Analyses used weights created by the National Center for Health Statistics that adjust for linkage eligibility.

**Results:**

Among 1050 adults in the study (41.6% aged ≥65 years; 70.1% female), 795 were receiving rental assistance at time of the NHANES interview (450 lived in project-based housing, and 345 had housing vouchers), and 255 received rental assistance within 2 years after the interview. Participants in project-based housing had lower HbA_1c_ levels compared with individuals in the waitlist group (β, −0.290; 95% CI, −0.599 to 0.020), but the difference was not significant. No significant differences in HbA_1c_ levels were found between those receiving housing vouchers and those in the waitlist group (β, 0.051; 95% CI, −0.182 to 0.284). Receiving project-based housing was associated with a reduced likelihood of uncontrolled diabetes (−3.7 percentage points; 95% CI, −7.0 to −0.0 percentage points) compared with being in the waitlist group.

**Conclusions and Relevance:**

In this cohort study of a nationally representative sample of US adults, living in project-based, federally subsidized housing was associated with a reduced likelihood of uncontrolled diabetes. The findings suggest that affordable housing programs may be associated with improved diabetes outcomes.

## Key Points

**Question:**

Is receiving housing rental assistance associated with glycated hemoglobin A_1c_ (HbA_1c_) levels among middle-aged and older US adults?

**Findings:**

In this cohort study of 1050 US adults aged 45 years or older, participants who were receiving federal rental assistance through project-based housing were less likely to have an HbA_1c_ level indicating uncontrolled diabetes compared with participants who would receive project-based housing within 2 years. Significant associations were not found for housing vouchers.

**Meaning:**

The findings suggest that rental assistance programs may be associated with improved diabetes outcomes among US adults.

## Teaser

This cohort study uses National Health and Nutrition Examination Survey data linked with US Department of Housing and Urban Development records to investigate whether receipt of federal housing rental assistance is associated with hemoglobin A_1c_ levels and related diabetes outcomes among US adults aged 45 years or older.

## Introduction

Exposure to unaffordable, unsafe, and unstable housing has negative consequences for health across the life course, with disproportionate effects among adults with low income and racial and ethnic minority individuals.^1,2^ Frequent moves and high rent burdens have been associated with an increased risk of diabetes and can make it more difficult to manage chronic conditions such as diabetes that require complex behaviors, adherence to medications, and regular health care visits.^3,4^ Programs that provide access to affordable and stable housing, such as rental assistance programs funded by the US Department of Housing and Urban Development (HUD), may be associated with reduced risk of diabetes and improved ability of adults to manage and control preexisting diabetes, with implications for long-term diabetes outcomes.^3^ However, a paucity of research exists on this topic, and the causal relationship is unclear.

During the Great Recession and mortgage crisis in 2008 to 2010 and in the subsequent years, the number of rent-burdened families in the US increased considerably, and homelessness increased among middle-aged and older adults.^5^ Current government policy solutions designed to improve the affordability of housing have not been sufficient to curb the rental crisis, and approximately 30 million individuals in need of rental assistance are unable to receive it given lengthy waiting periods.^6^ Specifically, underinvestment in project-based housing may be associated with increased economic and racial disparities in outcomes of diabetes and other chronic diseases, particularly because federal resources for rental assistance have increasingly emphasized housing vouchers instead of public housing.^7^ The nationwide housing affordability crisis has increasingly strained the ability of families with low income to obtain decent and stable housing; in 2019, approximately 83% of renters in the bottom income quintile spent at least 30% of their income on rent, and nearly three-fourths spent more than half.^8^ HUD rental assistance programs provide one of the few affordable options for this group by subsidizing tenant rent to 30% of income.^9^ Rental assistance is provided by HUD in 2 primary forms: project-based programs and tenant-based programs.^10^ Project-based housing includes public housing (owned by a public housing agency) and multifamily housing (multiple programs providing privately owned subsidized units), which typically provide units at affordable rates as part of a wholly or partially subsidized development.^9^ In contrast, tenant-based vouchers offer greater residential flexibility and mobility by allowing assisted families to rent a private-market unit and transfer their voucher to a new unit as desired.^11^ In 2020, HUD provided rental assistance to 4.6 million families,^10^ but demand for these programs largely outpaced supply. Fewer than 1 in 4 eligible families receive assistance, and most housing agencies maintain lengthy waiting lists that average 2 years.^6^ This supply shortage leaves millions of families with low income without secure housing. It also creates conditions that make it possible to examine how access to affordable housing may be associated with improved health.

Differences between project-based housing and vouchers suggest that it may be important to consider their association with health outcomes separately. Specifically, recent work suggests that short-term health benefits of rental assistance are greater for project-based housing than for vouchers and that there may be differences in the health benefits of rental assistance by race and ethnicity and by gender.^12^ Public and multifamily housing are associated with better self-reported health,^13^ fewer mental health symptoms,^14,15^ reduced risk of emergency department visits for asthma,^16^ and reduced alcohol and drug dependence.^14^ Other studies reported that both project-based housing and housing vouchers were associated with increased health care access,^17^ increased physical activity,^18^ and fewer missed school days owed to illness.^19^ To our knowledge, no quantitative studies have examined whether rental assistance is associated with improved glycemia and related diabetes outcomes, although qualitative evidence suggests that rental assistance may facilitate diabetes management.^20,21^

This study examined whether access to rental assistance programs is associated with levels of glycated hemoglobin A_1c_ (HbA_1c_). Hemoglobin A_1c_ levels reflect mean blood glucose levels over 3 months and are used to identify prediabetes (HbA_1c_ level, 5.7% to ≤6.5%), diagnose diabetes (HbA_1c_ level, >6.5%), and assess diabetes control (HbA_1c_ level, ≥9.0% [poor control]) (to convert HbA_1c_ to the proportion of total hemoglobin, multiply by 0.01). Higher HbA_1c_ levels have been associated with adverse health outcomes, including kidney disease, blindness, and cognitive decline.^22^ Diabetes is an important condition to study with respect to rental assistance because it disproportionately impacts adults with low income and racial and ethnic minority groups, who are at greater risk for housing insecurity.^23^ We used data that link national survey and biomarker measurements with administrative records from HUD on rental assistance participation and episodes during a 17-year period. We compared glycemic outcomes in 2 groups: adults receiving rental assistance and those who would receive rental assistance within the following 2 years, the mean waiting time for rental assistance.^10^ We examined associations of project-based housing programs vs housing vouchers with HbA_1c_ levels and considered differences by sex and race and ethnicity.

## Methods

### Data Sources

In this cohort study, we used data from the National Health and Nutrition Examination Survey (NHANES) linked with HUD administrative rental assistance records.^24^ Public-use NHANES data were obtained from the National Center for Health Statistics. NHANES is a nationally representative household survey that combines questionnaires with laboratory biomarker measurements. We used data from January 1999 to December 2016. The HUD administrative record contains longitudinal information on HUD rental assistance entry and exit and the type of housing program (project-based housing or voucher); these data were used to generate a record of rental assistance spells across the study period. Administrative records are necessary to overcome biases from self-reports of rental assistance participation.^25^ To adjust for potential bias, the National Center for Health Statistics created weights that account for linkage eligibility and nonresponse to make estimates representative of the civilian, noninstitutionalized US population.^24^ The Penn State institutional review board exempted the study from review and informed consent because it was not human participants research and the data were deidentified. The study followed the Strengthening the Reporting of Observational Studies in Epidemiology (STROBE) reporting guideline.

### Primary Outcome

In contrast to relying on self-reported diagnoses of diabetes, the NHANES Mobile Exam Center obtains laboratory serum samples for a subset of NHANES participants in each survey cycle. The primary outcome of the current study was HbA_1c_ level, the most widely used clinical measure to estimate mean blood glucose level, identify prediabetes (HbA_1c_ level, 5.7% to ≤6.5%), diagnose diabetes (HbA_1c_ level, >6.5%), and monitor diabetes control (HbA_1c_ level, ≥9% [poor glycemic control]). We used continuous levels of HbA_1c_ in our primary analysis. To better assess the association of rental assistance with diabetes management and control, in additional analyses, we examined uncontrolled diabetes (HbA_1c_ level, ≥9.0%) as the outcome. We also considered 2 additional dichotomous measures of HbA_1c_ that correspond to clinically relevant diabetes cut points: HbA_1c_ level of 5.7% to 6.5% (prediabetes) and greater than 6.5% (diabetes). Because the sample size of adults with diabetes was limited, we included adults regardless of diabetes diagnosis.

### Rental Assistance Status

HUD rental assistance status, including specific information on the housing program (project-based housing or housing vouchers), at the time of the NHANES interview was measured using the HUD administrative record. Given the strength of selection into rental assistance,^26,27^ we used the timing of entry into rental assistance to develop a control group of adults who were not receiving rental assistance but would enter rental assistance shortly after the NHANES interview. The waitlist comparison group included those who would enter assistance within 2 years, the mean time spent on a waiting list for HUD housing.^10^ The control group adjusted for unobserved characteristics that selected adults into rental assistance (eg, adverse health events, homelessness, or economic disadvantage). Although data on formal waiting lists were unavailable, the study design improved on a direct observation of waitlist status because all adults on a waiting list in the current analysis eventually received rental assistance. In contrast, many individuals on actual rental assistance waitlists do not obtain this resource owing to eligibility barriers.^28^ Respondents in the waitlist group were assigned to the first program they entered and were compared with current participants in that program. This waitlist design has been used in previous studies on the association of HUD housing with health^29,30^ and to address selection bias in studies of other policies.^31,32,33^

### Individual and Family Characteristics

We included the following covariates from NHANES: age (years), sex, race and ethnicity (Hispanic, non-Hispanic Black, non-Hispanic White, or other [included individuals of all other races and ethnicities; specific categories were not collected by NHANES]), educational level (less than high school, high school diploma, some college, or bachelor’s degree), and family income-to-poverty ratio (<50%, 50% to <100%, 100% to <200%, or ≥200% of the federal poverty level [FPL] or missing data). Because there may be geographic differences in housing supply and housing agency priorities, we adjusted for US state of residence. In addition, we adjusted for NHANES survey cycle (2-year periods during 1999-2016) to account for temporal changes in rental assistance and diabetes outcomes. The inclusion of these covariates adjusted for remaining sociodemographic and geographic differences between the current assistance and waitlist groups.

### Statistical Analysis

Data were analyzed in October 2021. We used linear regression to examine the association between rental assistance and HbA_1c_ levels, controlling for individual and family covariates, survey cycle, and state of residence. To evaluate whether the association differed by HUD housing program, we estimated models separately for adults in project-based housing and those receiving housing vouchers. Next, because diabetes risk varies by sex and race and ethnicity,^34^ we included interactions between rental assistance status and these characteristics. The sample size limited us from considering racial and ethnic groups other than Hispanic, non-Hispanic Black, and non-Hispanic White adults.

We used logistic regression to examine dichotomous HbA_1c_ level cutoffs (5.7%, 6.5%, and 9.0%). These models estimated the odds that an adult’s measured HbA_1c_ level was above each cutoff as a function of current rental assistance, individual and family covariates, survey cycle, and state of residence. All analyses were performed with Stata, version 17 (StataCorp LLC), used weights created by the National Center for Health Statistics that adjust for linkage eligibility, and accounted for the complex design of NHANES. The significance level was *P* < .05 using 1-tailed tests.

## Results

The linked data file included 19 914 linkage-eligible adults aged 45 years or older who were interviewed in NHANES from 1999 to 2016 and had no missing information on the covariates and outcomes. The analytical sample included 1050 adults who were either currently receiving rental assistance or in the waitlist group; 41.6% were aged 65 years or older, 70.1% were female, 14.5% were Hispanic, 36.4% were non-Hispanic Black, 42.6% were non-Hispanic White, and 6.4% were from other racial and ethnic groups. A total of 795 adults (4.0% of the linkage-eligible sample and 75.7% of the analytical sample) were currently receiving rental assistance; 450 were in project-based housing, and 345 were receiving housing vouchers. The remaining 255 respondents would enter rental assistance within 2 years of their interview.

Descriptive characteristics of the sample stratified by rental assistance status (current rental assistance or waitlist) are shown in Table 1. The group characteristics were similar. Among current recipients, 71.7% were female, 15.5% were Hispanic, 36.4% were non-Hispanic Black, 40.3% were non-Hispanic White, 7.8% were from other racial and ethnic groups, 72.6% had a high school education or less, and 56.1% had an income less than 100% of the FPL. Among those on a waitlist, 65.0% were female, 11.7% were Hispanic, 36.5% were non-Hispanic Black, 49.8% were non-Hispanic White, 2.0% were from other racial and ethnic groups, 71.3% had a high school education or less, and 43.8% had an income less than 100% of the FPL. There were significant differences between the current assistance and waitlist groups in housing program type, race and ethnicity, and family income-to-poverty ratio, although these differences were not significant after controlling for state of residence and survey year. eTables 1 and 2 in the Supplement show comparisons with adults who received no rental assistance during the period as well as program-specific descriptive characteristics.

**Table 1.** Descriptive Characteristics of 1050 Participants in the NHANES-HUD Sample by Linked Data File Rental Assistance Status From 1999 to 2016

Characteristic	Participants, %^a^		*P* value^d^
	Current rental assistance (n = 795)^b^	Waitlist (n = 255)^c^	
Rental assistance program			
Project-based housing	51.7	66.2	.002
Vouchers	48.3	33.8	
Sex			
Female	71.7	65.0	.17
Male	28.3	35.0	
Age group, y			
45-64	57.8	60.2	.62
≥65	42.2	39.8	
Race and ethnicity			
Hispanic	15.5	11.7	.02
Non-Hispanic			
Black	36.4	36.5	
White	40.3	49.8	
Other^e^	7.8	2.0	
Educational level			
Less than high school	47.4	40.9	.29
High school	25.3	30.4	
Some college	20.8	24.4	
Bachelor’s degree	6.6	4.3	
Family income-to-poverty ratio, % FPL			
<50	12.8	6.7	.01
50 to <100	43.3	37.1	
100 to <200	33.9	40.2	
≥200	4.8	9.7	
Missing	5.2	6.3	
HbA_1c_ level, %^f^			
<5.7	43.9	48.2	.45
5.7 to <6.5	35.2	33.2	
6.5 to <9.0	16.1	12.4	
≥9.0	4.8	6.2	

Abbreviations: FPL, federal poverty level; HbA_1c_, glycated hemoglobin A_1c_; HUD, US Department of Housing and Urban Development; NHANES, National Health and Nutrition Examination Survey.

SI conversion factor: To convert HbA_1c_ to the proportion of total Hb, multiply by 0.01.

^a^ The sample was limited to adults aged 45 years or older who received rental assistance at some point during the observation period from 1999 to 2016. Values are weighted to account for eligibility for NHANES-HUD linkage.

^b^ Receiving rental assistance at the time of the NHANES interview.

^c^ Not receiving assistance at the time of the NHANES interview but would enter assistance within 2 years of the interview.

^d^ *P* values shown are for a χ^2^ test of the difference between current recipients and individuals in the waitlist group.

^e^ Other included individuals of all other races and ethnicities; specific categories were not collected by the NHANES.

^f^ Hemoglobin A_1c_ is a common measure of blood glucose used to diagnose diabetes. An HbA_1c_ level of 5.7% to 6.5% corresponds to prediabetes, greater than 6.5% to diabetes, and 9.0% or greater to uncontrolled diabetes.

In regression models of the association between HbA_1c_ levels and rental assistance status (current vs waitlist) separately by housing program and adjusted for individual and family covariates, survey cycle, and state of residence, for housing vouchers (n = 435), HbA_1c_ levels were similar between current voucher recipients and adults in the waitlist group (β, 0.051; 95% CI, −0.182 to 0.284). For project-based housing (n = 615), current assistance recipients had lower HbA_1c_ levels compared with adults in the waitlist group (β, −0.290; 95% CI, −0.599 to 0.020), but the difference was not significant. Complete results are shown in eTable 3 in the Supplement.

Models in Table 2 and Table 3 assessed project-based housing (corresponding results for vouchers are shown in eTables 4 and 5 in the Supplement). Table 2 presents models including interactions of project-based housing with sex and race and ethnicity. In model 1, which examined differences between male and female participants, for males, project-based housing was not associated with a reduction in HbA_1c_ levels compared with the waitlist group (β, −0.454; 95% CI, −0.945 to 0.037). The interaction for sex was not statistically significant. Model 2 considered differences by race and ethnicity and showed that project-based housing was associated with a significant reduction in HbA_1c_ levels among non-Hispanic Black adults (β, −0.599; 95% CI, −1.132 to −0.065).

**Table 2.** Linear Regression Models of HbA_1c_ Levels as a Function of the Interactions of Project-Based Housing Assistance With Participants’ Sex and Race and Ethnicity^a^

	Model 1 (n = 615)^b^		Model 2 (n = 615)^c^	
	β (95% CI)	*P* value	β (95% CI)	*P* value
Currently receiving rental assistance	−0.454 (−0.945 to 0.037)	.07	−0.599 (−1.132 to −0.065)	.03
Female	−0.237 (−0.753 to 0.280)	.36	NA	NA
Race and ethnicity				
Hispanic	NA	NA	−0.934 (−1.662 to −0.206)	.01
Non-Hispanic White	NA	NA	−0.800 (−1.400 to −0.200)	.01
Other^d^	NA	NA	−0.820 (−1.713 to 0.074)	.07
Interactions				
Female × current assistance	0.253 (−0.391 to 0.897)	.43	NA	NA
Hispanic × current assistance	NA	NA	0.749 (−0.165 to 1.663)	.11
White × current assistance	NA	NA	0.380 (−0.324 to 1.084)	.28
Other race and ethnicity × current assistance	NA	NA	0.778 (−0.200 to 1.756)	.12

Abbreviations: HbA_1c_, glycated hemoglobin A_1c_; HUD, US Department of Housing and Urban Development; NA, not applicable; NHANES, National Health and Nutrition Examination Survey.

^a^ Linked data from NHANES and HUD from 1999 to 2016 were used. Models assessed interactions with continuous HbA_1c_ levels among adults aged 45 years or older. All models accounted for the complex survey design of NHANES, were weighted to reflect eligibility for linkage to the HUD record, and were adjusted for individual and family covariates (sex, age, race and ethnicity, educational level, and family income-to-poverty ratio), NHANES survey year cycle, and state of residence.

^b^ Model 1 included an interaction between rental assistance status and sex. The main effect for current assistance in model 1 refers to males, the reference group.

^c^ Model 2 included an interaction between rental assistance status and race and ethnicity. The main effect for current assistance in model 2 refers to non-Hispanic Black adults, the reference group.

^d^ Other included individuals of all other races and ethnicities; specific categories were not collected by the NHANES.

**Table 3.** Mean Marginal Associations of Project-Based Rental Assistance With Dichotomous HbA_1c_ Level Cutoffs

	Difference, percentage points (95% CI)^a^		
	HbA_1c_ level 5.7% to ≤6.5% (n = 615)	HbA_1c_ level >6.5% (n = 615)	HbA_1c_ level ≥9.0% (n = 615)
Currently receiving rental assistance	−3.9 (−14.7 to 6.8)	−3.4 (−14.4 to 7.6)	−3.7 (−7.0 to −0.0)^b^
Reference group mean of dependent variable	55.8	22.1	6.9

Abbreviations: HbA_1c_, glycated hemoglobin A_1c_; HUD, US Department of Housing and Urban Development; NHANES, National Health and Nutrition Examination Survey.

SI conversion factor: To convert HbA_1c_ to the proportion of total Hb, multiply by 0.01.

^a^ Linked data from NHANES and HUD from 1999 to 2016 were used. Values indicate the mean percentage point difference in the probability of being above the HbA_1c_ cutoff when receiving current assistance vs being in the waitlist group. All models accounted for the complex survey design of NHANES, were weighted to reflect eligibility for linkage to the HUD record, and were adjusted for individual and family covariates (sex, age, race and ethnicity, educational level, and family income-to-poverty ratio), NHANES survey year cycle, and state of residence. An HbA_1c_ level of 5.7% to 6.5% corresponds to prediabetes, greater than 6.5% to diabetes, and 9.0% or greater to uncontrolled diabetes.

^b^ *P* < .05.

Table 3 presents mean marginal effects^35^ for dichotomous measures of HbA_1c_ that correspond to clinically relevant cut points, including those for prediabetes (HbA_1c_ level, 5.7% to ≤6.5%), diabetes (HbA_1c_ level, >6.5%), and poor control of diabetes (HbA_1c_ level, ≥9.0%). Currently living in project-based housing was associated with a reduction in the probability of having uncontrolled diabetes. The probability was 3.7 percentage points (95% CI, 0.0-7.0 percentage points) lower for project-based housing recipients compared with the mean 6.9% prevalence of uncontrolled diabetes in the waitlist group. Project-based housing was not associated with a reduced likelihood of reaching HbA_1c_ thresholds that are typically used to diagnose prediabetes and diabetes. Consistent with the continuous HbA_1c_ measure, housing vouchers were not associated with a reduced likelihood of elevated HbA_1c_ levels (eTable 5 in the Supplement).

## Discussion

In this cohort study of a national representative sample of US adults aged 45 years or older, participants receiving assistance through project-based housing had lower HbA_1c_ levels than did adults who were scheduled to receive assistance within 2 years, but the difference was not statistically significant. The differences were largest for non-Hispanic Black adults. Of importance, project-based housing assistance was associated with a reduced likelihood of poor glycemic control (HbA_1c_ level, ≥9.0%), suggesting that rental assistance has clinically significant implications for the risk of adverse diabetes outcomes. To our knowledge, this is the first evidence to suggest that project-based housing is associated with reduced likelihood of uncontrolled diabetes. We found no significant associations between receiving housing vouchers and HbA_1c_ level.

Access to rental assistance may be associated with improved diabetes self-management and control by reducing housing costs that can compete with diabetes-related expenses, such as those related to healthy food and medications. Access to affordable housing may also be associated with improved diabetes control by reducing housing stress and providing both stability and autonomy that allow individuals to prioritize their diabetes care and to maintain consistent self-management routines.^20,21^ Access to stable housing arrangements may be associated with improved mental health and reduced distress,^12^ which can increase feelings of self-efficacy regarding managing chronic disease.^36,37^ Prior research indicated that recipients of rental assistance experienced greater subjective stability, fewer moves to new housing, and greater housing satisfaction than did those in the waitlist group.^38^ However, our findings suggest that the benefits of rental assistance for diabetes depend on the specific housing program.

Although differences were not significant, individuals in project-based housing programs had reduced HbA_1c_ levels compared with those in the waitlist group; HbA_1c_ levels were similar in those receiving housing vouchers and those in the waitlist group. These findings are largely consistent with existing work using self-reported outcomes.^16,29^ Residents of public housing reported a longer length of residence than did voucher holders, and this greater housing stability may be associated with improved diabetes management.^39,40^ Project-based housing may also facilitate the development of strong social ties and informal networks of support.^41,42^ Prior work found that Black residents of public housing reported significantly greater access to neighborhood social support than did recipients of other forms of rental assistance.^43^

In contrast, housing vouchers often require moves to unfamiliar residential environments, which can disrupt social networks. In addition, housing vouchers may more often be used in lower-density neighborhoods or municipalities, where access to transportation may be reduced and physical activity or access to healthy food and high-quality care may be impeded. Vouchers may also provide less of an affordability benefit because they often do not include energy subsidies and households may spend up to 40% of their income on rent. Results from the Moving To Opportunity study,^35^ which provided public housing residents with vouchers to relocate to low-poverty areas, found that adults in the experimental voucher group, which restricted voucher use to low-poverty areas and provided support to access those areas, experienced reductions in rates of obesity and blood glucose levels compared with the control group. However, the Moving To Opportunity study did not test the effects of vouchers vs no rental assistance and was focused on the effects of neighborhood poverty.^44^

We found no association between rental assistance and glycemic control by sex. Limited research exists on sex differences in the association between rental assistance and health among adults. Men may experience greater improvements in housing stability associated with rental assistance than women do. Men may be at greater risk of homelessness, living with multiple families in a single residence, or other adverse housing situations in the absence of subsidized housing^45^ and may experience more health problems associated with housing instability than women do.

Our results indicated an association between rental assistance and levels of glycemic control among non-Hispanic Black adults. Our findings for Black adults are consistent with other studies demonstrating that safety net programs, including rental assistance, often disproportionately benefit Black individuals in the US.^46,47^ The improvements in housing circumstances may also be greater for Black adults than for White or Hispanic adults owing to ongoing structural racism that has constrained housing options for Black individuals in the US and supported housing opportunities for White individuals in the US.^48^ As a result, Black individuals in the US are more likely to experience rental cost burdens, evictions, and homelessness.^8^ For many Black families, the networks in public and other housing projects may also represent an important source of support that can facilitate not only access to care, the ability to adhere to complex medical regimens, and access to healthy foods but also resistance to racism.^49^

### Limitations

This study has limitations. First, we were unable to examine changes in HbA_1c_ levels in individuals over time and instead compared adults who differed only based on whether they had entered HUD housing. Future longitudinal research is important to evaluate whether the timing of entry into rental assistance is associated with HbA_1c_ levels. Second, our approach was not a randomized experiment and could not capture changes that occurred alongside entry into rental assistance that may have been associated with our outcomes. In addition, public housing agencies may prioritize certain groups for assistance (eg, people with disabilities, families experiencing homelessness). However, our findings would likely be more conservative if individuals with poor health owing to diabetes were prioritized and were therefore more likely to be current recipients of assistance. Third, the statistical power was limited to consider project-based housing programs individually (eTable 6 in the Supplement). Statistical power also limited our ability to assess the impact of rental assistance within subgroups of adults with preexisting diagnosed diabetes or prediabetes. Fourth, we were unable to consider other potential mediators of the association between rental assistance and HbA_1c_ levels, including housing quality, diet, physical activity, or other chronic disease indicators. This is a fruitful area for future research.

## Conclusions

In this cohort study of a nationally representative sample of US adults aged 45 years or older, receipt of rental assistance for project-based housing was associated with a reduced likelihood of uncontrolled diabetes compared with receiving housing vouchers or being in the waitlist group for rental assistance. These findings suggest that the mobility benefits of housing vouchers may need to be balanced against the increasing evidence for the health benefits of project-based housing.

## Notes

Supplement. **eTable 1.** Descriptive Characteristics of NHANES-HUD Sample by Rental Assistance Status 1999-2016 (N = 19 914) **eTable 2.** HUD Program-Specific Descriptive Characteristics of NHANES-HUD Sample by Rental Assistance Status 1999-2016 (N = 1050) **eTable 3.** Linear Models Predicting Hemoglobin A_1c_ (%) as a Function of Rental Assistance by Housing Program, NHANES-HUD 1999-2016 **eTable 4.** Linear Models Predicting A_1c_ (%) as a Function of Voucher Assistance With Gender and Race and Ethnicity Interactions, NHANES-HUD 1999-2016 **eTable 5.** Marginal Effects of Voucher Assistance on Dichotomous A_1c_ Cutoffs, NHANES-HUD 1999-2016 **eTable 6.** Linear Models Predicting Hemoglobin A_1c_ (%) as a Function of Rental Assistance for Public Housing and Multifamily Housing, NHANES-HUD 1999-2016
