# Supplementary material for: Association Between Rental Assistance Programs and Hemoglobin A1c Levels Among US Adults
Source: JAMA Netw Open. 2022 Jul 20;5(7):e2222385. doi: 10.1001/jamanetworkopen.2022.22385 (PMC9301513; doi:10.1001/jamanetworkopen.2022.22385)
Supplement: Supplement. — eTable 1. Descriptive Characteristics of NHANES-HUD Sample by Rental Assistance Status 1999-2016 (N = 19 914) eTable 2. HUD Program-Specific Descriptive Characteristics of NHANES-HUD Sample by Rental Assistance Status 1999-2016 (N = 1050) eTable 3. Linear Models Predicting Hemoglobin A1c (%) as a Function of Rental Assistance by Housing Program, NHANES-HUD 1999-2016 eTable 4. Linear Models Predicting A1c (%) as a Function of Voucher Assistance With Gender and Race and Ethnicity Interactions, NHANES-HUD 1999-2016 eTable 5. Marginal Effects of Voucher Assistance on Dichotomous A1c Cutoffs, NHANES-HUD 1999-2016 eTable 6. Linear Models Predicting Hemoglobin A1c (%) as a Function of Rental Assistance for Public Housing and Multifamily Housing, NHANES-HUD 1999-2016 [file jamanetwopen-e2222385-s001.pdf]

Supplementary Online Content

Fenelon A, Lipska KJ, Denary W, et al. Association between rental assistance programs and hemoglobin A1c levels among US adults. *JAMA Netw Open*. 2022;5(7):e2222385. doi:10.1001/jamanetworkopen.2022.22385

eTable 1. Descriptive Characteristics of NHANES-HUD Sample by Rental Assistance Status 1999-2016 (N = 19 914)
eTable 2. HUD Program-Specific Descriptive Characteristics of NHANES-HUD Sample by Rental Assistance Status 1999-2016 (N = 1050)
eTable 3. Linear Models Predicting Hemoglobin A_{1c} (%) as a Function of Rental Assistance by Housing Program, NHANES-HUD 1999-2016
eTable 4. Linear Models Predicting A_{1c} (%) as a Function of Voucher Assistance With Gender and Race and Ethnicity Interactions, NHANES-HUD 1999-2016
eTable 5. Marginal Effects of Voucher Assistance on Dichotomous A_{1c} Cutoffs, NHANES-HUD 1999-2016
eTable 6. Linear Models Predicting Hemoglobin A_{1c} (%) as a Function of Rental Assistance for Public Housing and Multifamily Housing, NHANES-HUD 1999-2016

This supplementary material has been provided by the authors to give readers additional information about their work.

© 2022 Fenelon A et al. *JAMA Network Open*.

eTable 1: Descriptive Characteristics of NHANES-HUD Sample by Rental Assistance Status 1999-2016 (N = 19 914)			
	Current[a]	Pseudo-waitlist[b]	No Rental Assistance[c]
n	795	255	18,864
Rental Assistance Program**			
Project-Based Housing	0.517	0.662	NA
Vouchers	0.483	0.338	NA
Female**	0.717	0.650	0.523
Age Groups**			
45-64	0.578	0.602	0.673
65+	0.422	0.398	0.327
Race/Ethnicity**			
non-Hispanic White	0.403	0.498	0.765
non-Hispanic Black	0.364	0.365	0.089
non-Hispanic Other	0.078	0.020	0.054
Hispanic	0.155	0.117	0.092
Education**			
Less than High School	0.474	0.409	0.180
High School	0.253	0.304	0.244
Some College	0.208	0.244	0.295
Bachelor's Degree	0.066	0.043	0.281
Family Poverty Status**			
Below 50% FPL	0.128	0.067	0.022
50-99% of FPL	0.433	0.371	0.067
100-199% of FPL	0.339	0.402	0.184
200%+ of FPL	0.048	0.097	0.677
Missing	0.052	0.063	0.051

Notes: Sample is limited to adults ages 45+. FPL=Federal Poverty Line.
[a]Receiving rental assistance at interview.
[b]Not receiving assistance at interview, but will enter assistance within 2 years of interview.
[c]Not receiving rental assistance at interview or within 2 years 1999 and 2016.
Source: NHANES-HUD linked data file 1999-2016
†p<0.1 *p<0.05 **p<0.01 – Chi-Square test of differences across groups

© 2022 Fenelon A et al. *JAMA Network Open*.

eTable 2: HUD Program-Specific Descriptive Characteristics of NHANES-HUD Sample by Rental Assistance Status 1999-2016 (N = 1050)				
	Vouchers		Project-Based Housing	
	Current[a]	Pseudo-waitlist[b]	Current[a]	Pseudo-waitlist[b]
n	345	90	450	165
Mean Assistance Duration (Years)	4.6		4.5	
Female	0.701	0.713	0.732	0.618
Age Groups				
45-64	0.703	0.745	0.446	0.512
65+	0.297	0.255	0.554	0.488
Race/Ethnicity	*		*	
non-Hispanic White	0.431	0.283	0.377	0.608
non-Hispanic Black	0.343	0.532	0.384	0.279
non-Hispanic Other	c	c	0.113	0.027
Hispanic	c	c	0.125	0.086
Education				
Less than High School	0.450	0.374	0.495	0.427
High School	0.254	0.248	0.252	0.333
Some College	c	c	0.180	0.192
Bachelor's Degree	c	c	0.073	0.049
Family Poverty Status*				
Below 50% FPL	0.153	0.114	0.105	0.043
50-99% of FPL	0.445	0.364	0.422	0.375
100-199% of FPL	0.315	0.377	0.361	0.414
200%+ of FPL	0.035	0.113	0.060	0.089
Missing	0.052	0.032	0.052	0.079

Notes: Sample is limited to adults ages 45+ who receive rental assistance at some point during the observation period 1999-2016. FPL=Federal Poverty Line.

[a]Receiving rental assistance at interview.

[b]Not receiving assistance at interview, but will enter assistance within 2 years of interview.

Source: NHANES-HUD linked data file 1999-2016

[c]Value suppressed due to small sample size to avoid disclosure risk.

†p<0.1 *p<0.05 **p<0.01 – Chi-Square test of differences between current and pseudo-waitlist by program

© 2022 Fenelon A et al. *JAMA Network Open*.

eTable 3: Linear Models Predicting Hemoglobin A_{1c} (%) as a Function of Rental Assistance by Housing Program, NHANES-HUD 1999-2016		
	A1c (%)	
	Housing Voucher	Project-Based Housing
Rental Assistance Status		
Current Assistance	0.051 (-0.182,0.284)	-0.290 (-0.599,0.020)†
Age in years	0.004 (-0.008,0.016)	0.008 (0.000,0.017)*
Female	-0.225 (-0.580,0.130)	-0.060 (-0.262,0.141)
Race/Ethnicity		
Non-Hispanic White (ref.)		
Non-Hispanic Black	-0.110 (-0.316,0.096)	0.516 (0.280,0.751)***
Non-Hispanic Other	-0.241 (-1.292,0.809)	0.371 (0.074,0.669)*
Hispanic/Latino	-0.152 (-0.487,0.182)	0.167 (-0.119,0.453)
Education		
Less than High School (ref.)		
High School	0.130 (-0.208,0.469)	-0.375 (-0.674,-0.075)*
Some College	0.005 (-0.309,0.317)	-0.388 (-0.632,-0.143)**
Bachelor's Degree	0.123 (-0.586,0.831)	-0.127 (-0.637,0.384)
Family Poverty Status		
Below 50% FPL (ref.)		
50-99% of FPL	0.183 (-0.266,0.631)	0.334 (0.124,0.545)**
100-199% of FPL	-0.222 (-0.691,0.247)	0.321 (0.034,0.608)*
200%+ of FPL	-0.380 (-0.884,0.125)	0.592 (0.041,1.144)*
Missing	-0.416 (-1.159,0.327)	-0.134 (-0.457,0.430)
State and Year Fixed Effects	Yes	Yes
N	435	615

Note: Models predict continuous hemoglobin A_{1c} among adults ages 45+. All models account for the complex survey design of NHANES and are weighted to reflect eligibility for linkage to the HUD record. 95% confidence intervals in parentheses.

[a]Value suppressed due to small sample size to avoid disclosure risk.

†p<0.1 *p<0.05 **p<0.01

© 2022 Fenelon A et al. *JAMA Network Open*.

eTable 4: Linear Models Predicting A_{1c} (%) as a Function of Voucher Assistance With Gender and Race and Ethnicity Interactions, NHANES-HUD 1999-2016		
	A1c (%)	
	Housing Voucher	
	Model 1	Model 2
Rental Assistance Status		
Pseudo-waitlist (ref.)		
Current Assistance	0.545 (0.034,1.056)*	-0.007 (-0.345,0.330)
Female	0.355 (-0.083,0.793)	
Race/Ethnicity		
non-Hispanic white		0.006 (-0.686.0.698)
non-Hispanic Black (ref.)		
Other		a
Hispanic		-0.257 (-0.841,0.327)
Interactions		
Female x Current	-0.721 (-1.385,-0.058)*	
White x Current		0.136 (-0.571,0.844)
Other x Current		a
Hispanic x Current		0.282 (-0.535,1.100)
Sociodemographic Controls	Yes	Yes
State Fixed Effects	Yes	Yes
N	435	435

Note: Models predict continuous hemoglobin A_{1c} among adults ages 45+. All models account for the complex survey design of NHANES and are weighted to reflect eligibility for linkage to the HUD record. Model 1 includes an interaction between rental assistance status and gender. Model 2 includes an interaction between rental assistance status and race/ethnicity. All models adjust for individual and family covariates (gender, age, race/ethnicity, education level, family income-to-poverty ratio), NHANES survey year cycle, and state of residence. 95% confidence intervals in parentheses.
[a]Value suppressed due to small sample size to avoid disclosure risk.
†p<0.1 *p<0.05 **p<0.01

© 2022 Fenelon A et al. *JAMA Network Open*.

eTable 5: Marginal Effects of Voucher Assistance on Dichotomous A_{1c} Cutoffs, NHANES-HUD 1999-2016			
	Housing Voucher		
	A1c ≥ 5.7	A1c ≥ 6.5	A1c ≥ 9.0
Rental Assistance Status			
Pseudo-waitlist (ref.)			
Current Assistance	-6.0	2.5	2.7
95% CI	(-16.6,4.6)	(-6.9,11.8)	(-1.0,6.4)
Reference Group Mean of Dependent Variable	62.5	19.3	4.0
Sociodemographic Controls	Yes	Yes	Yes
State Fixed Effects	Yes	Yes	Yes
N	435	435	435

Note: Models predict likelihood of having hemoglobin A_{1c} above select cutoffs. A_{1c} >5.7% corresponds to prediabetes, 6.5% to diabetes, and 9.0% to uncontrolled diabetes. Values shown indicate average percentage point difference in the probability of being above the cutoff when receiving current assistance vs. pseudo-waitlist. All models account for the complex survey design of NHANES and are weighted to reflect eligibility for linkage to the HUD record. All models adjust for individual and family covariates (gender, age, race/ethnicity, education level, family income-to-poverty ratio), NHANES survey year cycle, and state of residence. 95% confidence intervals in parentheses.
†p<0.1 *p<0.05 **p<0.01

© 2022 Fenelon A et al. *JAMA Network Open*.

eTable 6: Linear Models Predicting Hemoglobin A_{1c} (%) as a Function of Rental Assistance for Public Housing and Multifamily Housing, NHANES-HUD 1999-2016		
	A1c (%)	
	Public Housing	Multifamily Housing
Rental Assistance Status		
Pseudo-waitlist (ref.)		
Current Assistance	-0.380 (-0.775,0.015)†	-0.145 (-0.478,0.188)
Sociodemographic Controls	Yes	Yes
State Fixed Effects	Yes	Yes
N	236	379

Note: Models predict continuous hemoglobin A_{1c} among adults ages 45+. All models account for the complex survey design of NHANES and are weighted to reflect eligibility for linkage to the HUD record. All models adjust for individual and family covariates (gender, age, race/ethnicity, education level, family income-to-poverty ratio), NHANES survey year cycle, and state of residence. Coefficients compare current assistance to the pseudo-waitlist group for each housing program. 95% confidence intervals in parentheses.
†p<0.1 *p<0.05 **p<0.01

© 2022 Fenelon A et al. *JAMA Network Open*.
